# Aspirin-triggered DHA metabolites inhibit angiogenesis

**DOI:** 10.3389/fphar.2025.1524980

**Published:** 2025-02-25

**Authors:** M. Vara-Messler, L. Trevisi, E. Zulato, G. E. Ramaschi, P. Risé, C. Pinna, S. Indraccolo, A. Sala, C. Bolego

**Affiliations:** ^1^ Department of Pharmaceutical and Pharmacological Sciences, University of Padova, Padova, Italy; ^2^ Pole of Pharmacology and Therapeutics (FATH), Institut de Recherche Expérimentale et Clinique (IREC), Université Catholique de Louvain (UC Louvain), Brussels, Belgium; ^3^ Basic and Translational Oncology Unit, Istituto Oncologico Veneto IOV-IRCCS, Padova, Italy; ^4^ Department of Pharmaceutical Sciences, University of Milan, Milan, Italy; ^5^ Department of Surgery Oncology and Gastroenterology, University of Padova, Padova, Italy

**Keywords:** angiogenesis, docosahexaenoic acid (DHA), aspirin (ASA), 17(R)-hydroxy-docosahexaenoic acid (17(R)-HDHA), human umbilical vein endothelial cells (HUVEC)

## Abstract

**Background and aim:**

Blood vessels supply oxygen, nutrients and provide gateways for immune surveillance. Since this network nourishes all tissues, vessel abnormalities contribute to many diseases, such as cancer. One of the potential targets for Docosahexaenoic Acid (DHA) in cancer is suppressing angiogenesis, a process of new blood vessel formation within tumors. In addition, aspirin (ASA) has antineoplastic effects that may be mediated, at least in part, by metabolites derived from acetylated COX-2. We aimed at determining the effect of DHA as well as its metabolites in angiogenesis, using *in vitro* as well as *in vivo* models.

**Methods:**

Endothelial cell (EC) proliferation, motility and capillary-like tube formation were determined by MTT, wound healing, Boyden and Matrigel assays, respectively. In vivo angiogenesis was measured by the Matrigel sponge model in mice. The biosynthesis of proresolving lipid mediators by ECs was determined by LC-MS-MS.

**Results and conclusion:**

DHA, but not arachidonic acid (AA), at concentrations consistent with those reached in blood after fish oil supplementation, decreased EC migration in a time- and concentration-dependent manner. Pretreatment with ASA modulated cell migration already after 24 h, while both DHA and ASA decreased migration at longer incubation times without affecting viability. 17-hydroxy-DHA was detected upon incubation with DHA, and increased amounts were observed upon combined treatment with DHA and ASA, an increase that was associated to a synergic effect on EC migration. 17(R)-hydroxy-DHA (17R-HDHA), the metabolite resulting from acetylated COX-2 activity of DHA, reduced EC migration in a concentration-dependent manner. DHA in the presence of ASA, as well as 17R-HDHA, also reduced EC tube formation. These results were confirmed *in vivo* where both 17R-HDHA or its downstream metabolite 17RResolvinD1 were able to decrease microvessels density in a Matrigel sponge model. Overall, we demonstrated that DHA in the presence of ASA-dependent acetylation of COX-2 showed increased antiangiogenic effects, possibly resulting from its conversion to its hydroxylated derivatives.

## 1 Introduction

Angiogenesis is a tightly regulated process occurring through dynamic functions of endothelial cells including migration, proliferation and formation of capillary-like structures. The angiogenic process is a necessary step in tumour growth and metastasis (Folkman, 1995), but is also a key feature of many pathological conditions such as chronic inflammatory disease, supporting the occurrence of a link between cancer and unresolved inflammation (Fishbein et al., 2020; Fishbein et al., 2021).

Angiogenesis is affected by several pro-angiogenic growth factors and cytokines, but a growing body of evidence is suggesting a potential role for fatty acids (He et al., 2023). Ω-6 and ω-3 polyunsaturated fatty acids (PUFAs) can affect angiogenesis through multiple and opposite mechanisms including their oxidation and the formation of PUFA-derived metabolites (Frömel et al., 2022; Kang and Liu, 2013; Quinlivan et al., 2024). PUFAs are substrates for enzymes such as cyclooxygenases (COXs), lipoxygenases (LOXs) and cytochrome P450 (CYP450s) that generate potent bioactive lipid mediators (Calder, 2020; Serhan et al., 2020). In particular, several COX-2- and/or COX-1-derived arachidonic acid (AA, 20:4 ω-6) metabolites promote angiogenesis (Salvado et al., 2012), whereas ω-3 PUFAs such as docosahexaenoic acid (DHA, 22:6 ω-3) have anti-angiogenic and anti-tumor properties (Kelly et al., 2024; Rose and Connolly, 1999), by serving as alternative substrates to generate ω-3 lipid mediators endowed with anti-inflammatory and pro-resolution activities (Gilligan et al., 2019; Quinlivan et al., 2024) and suppressing the formation of several pro-angiogenic factors (Spencer et al., 2009). Of note, DHA metabolites generated by LOXs mainly expressed in inflammatory cells have been shown to have a beneficial role in pathological retinal angiogenesis (Connor et al., 2007; Maisto et al., 2020) as well as in cancer-associated angiogenesis (Prevete et al., 2017; Sulciner et al., 2018a; Sulciner et al., 2018b).

Epidemiological studies showed that aspirin has the unique ability to reduce the risk of several cancers (Elwood et al., 2024; Skriver et al., 2024) but the mechanisms underlying this effect are only partially understood (Patrignani and Patrono, 2018). The pharmacological activity of aspirin (ASA) is linked to the irreversible acetylation of COXs, but whereas COX-1 acetylation results in the loss of enzymatic activity, ASA-acetylated COX-2 is unable to generate prostanoids but remains active as a 15(R)-lipoxygenase (Lecomte et al., 1994; Mancini et al., 1994), and has been shown to contribute to the biosynthesis of anti-inflammatory and pro-resolving lipid mediators known as aspirin triggered (AT)-lipoxins (Clària and Serhan, 1995). In addition to metabolizing AA, 15-lipoxygenases enzymatic activities can easily convert DHA into 17-hydroxy-DHA (Dobson et al., 2013), and treatment with ASA and DHA resulted in the novel formation of 17-hydroxy-DHA *in vivo* in mice (Serhan et al., 2002). 17(R) hydroxy-DHA (17R-HDHA) generated by ASA-acetylated COX-2 from DHA, can be further oxygenated by 5-LOX in inflammatory cells through transcellular metabolism, resulting in the formation of 17(R)-resolvin D1 (17R-RvD1), also referred to as Aspirin Triggered-resolvin D1 (Serhan et al., 2002). Aspirin-Triggered lipid metabolites belong to the superfamily of specialized pro-resolving lipid mediators (SPMs) (Serhan et al., 2020), and a growing body of evidence suggests that SPMs supplementation may be beneficial in several diseases, including cancer, by promoting resolution over time of disease-associated inflammation (Serhan and Levy, 2018). Gilligan et al. recently showed that the production of AT-SPMs (17R-RvD1 and AT-LXA_4_) may contribute to the antitumor activity of aspirin by promoting macrophage phagocytosis of tumor cell debris and functionally antagonizing macrophage secretion of proinflammatory cytokines (Gilligan et al., 2019). Given that inflammation and angiogenesis are strictly related processes (Mantovani et al., 2008; Trenti et al., 2018), AT-metabolites may affect angiogenesis, thus contributing to explain their pro-resolving potential in pathological conditions characterized by sustained low-grade phlogosis, such as cancer. The direct *in vitro* antiangiogenic effect of stable AA-derived AT-SPMs such as 15(R)-lipoxin A_4_ has been well characterized (Cezar-De-Mello et al., 2006; Cezar-De-Mello et al., 2008), but whether DHA metabolites from ASA-acetylated COX-2, and in particular 17R-HDHA, may affect angiogenesis is still substantially unexplored, with evidence available focusing on 17R-RvD1 (Maisto et al., 2020).

Based on this background, we tested (a) the antiangiogenic activity of PUFAs (AA or DHA) or aspirin alone or in combination, and (b) the effect of specific Aspirin Triggered-metabolites in *in vitro* and *in vivo* models of angiogenesis.

## 2 Results

### 2.1 Effects of DHA and AA on HUVEC viability and migration

**FIGURE 1:**
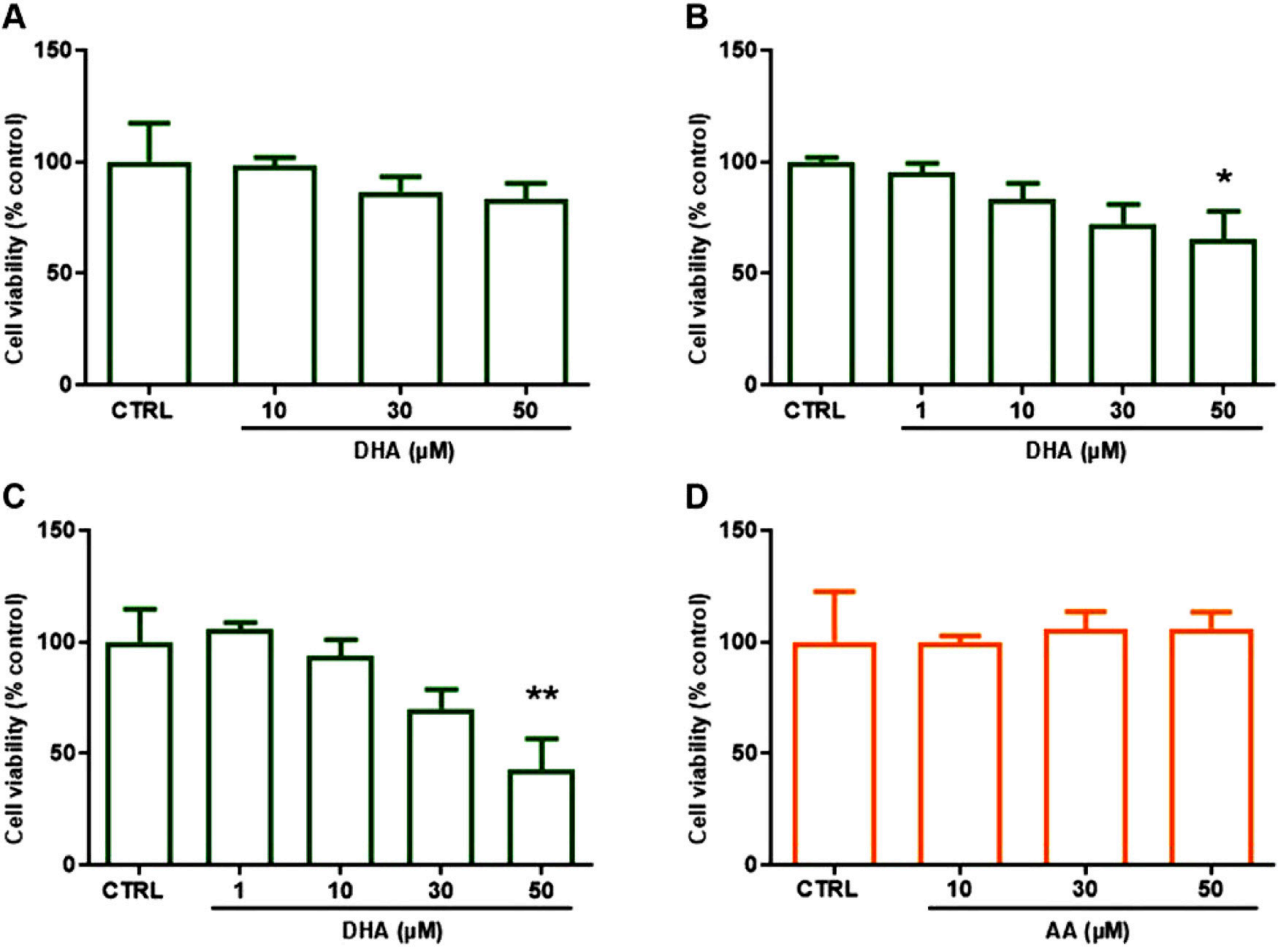
Effects of DHA (ω-3) and AA (ω-6) on HUVEC viability after 24–72 h. HUVECs were grown in 96-well plates and incubated in complete culture medium with DHA (1–50 µM) for **(A)** 24, **(B)** 48, **(C)** 72 h or **(D)** with AA (10–50 µM) for 48 h. Cell viability was assessed by MTT assay and expressed as % of control (untreated cells, CTRL). Bars show the mean ± SEM of 3 **(A)** or 4 **(B–D)** independent experiments performed in quadruplicate. One-way analysis of variance followed by Dunnett’s *post hoc* test: *p < 0.05, **p < 0.01 vs. control.

We first evaluated the effects of DHA on human umbilical vein endothelial cells (HUVECs) viability and migration at concentrations consistent with those reached in blood after fish oil supplementation (Yurko-Mauro et al., 2015). HUVEC viability was not significantly affected by treatment with 1–30 µM DHA for up to 72 h (Figures 1A–C), while 50 µM DHA, significantly decreased cell viability upon incubation for 48 h or 72 h (Figures 1B, C). Similarly, treatment of HUVECs with 1–50 µM AA for 48 h did not significantly affect cell viability (Figure 1D).

**FIGURE 2:**
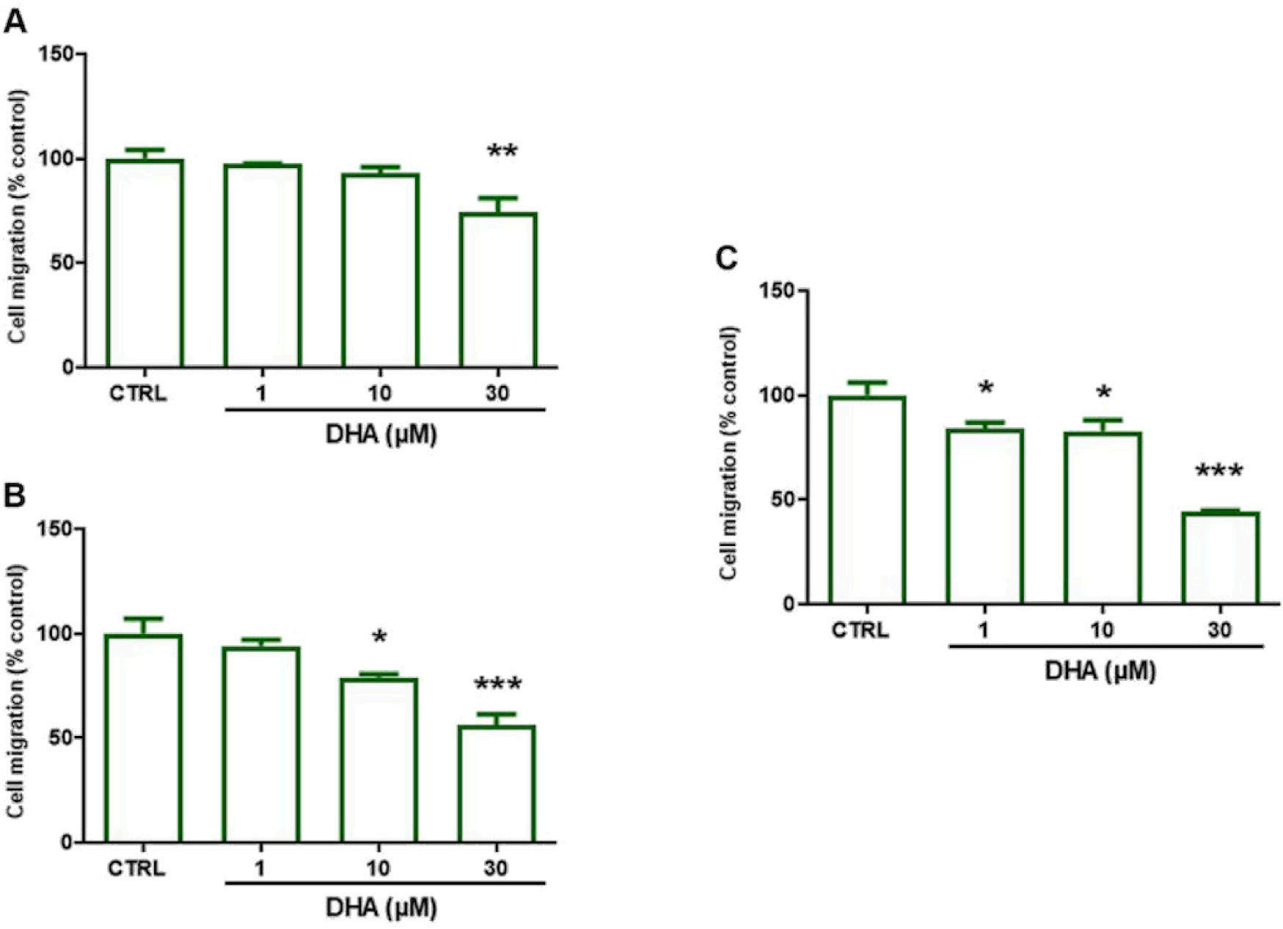
Effect of DHA on HUVEC migration after 24–72 h. HUVECs were grown in 24 well plates and the assay was performed in confluent cells. Cells were pre-treated with DHA (1–30 µM) for: **(A)** 24, **(B)** 48 or **(C)** 72 h. Thereafter, monolayers were wounded (t0), washed and treated as above for 16 h (t16). For each experimental condition 3 images were taken at t0 and t16; wound closure was calculated as described in Methods. Data are reported as % of control (untreated cells, CTRL). Bars show the mean ± SEM of 3 independent experiments. One-way analysis of variance followed by Dunnett’s *post hoc* test. *p < 0.05, **p < 0.01, ***p < 0.001 vs. control.

Then, in order to evaluate the effect of DHA on collective HUVEC migration, we used a wound healing assay. As shown in Figure 2, treatment with DHA time- and dose-dependently decreased HUVEC collective migration when compared to untreated control cells, with significant effects already observed at the concentration of 1 µM DHA after 72 h pre-treatment (Figure 2C).

### 2.2 DHA in the presence of ASA decreased endothelial cell migration without affecting viability

**FIGURE 3:**
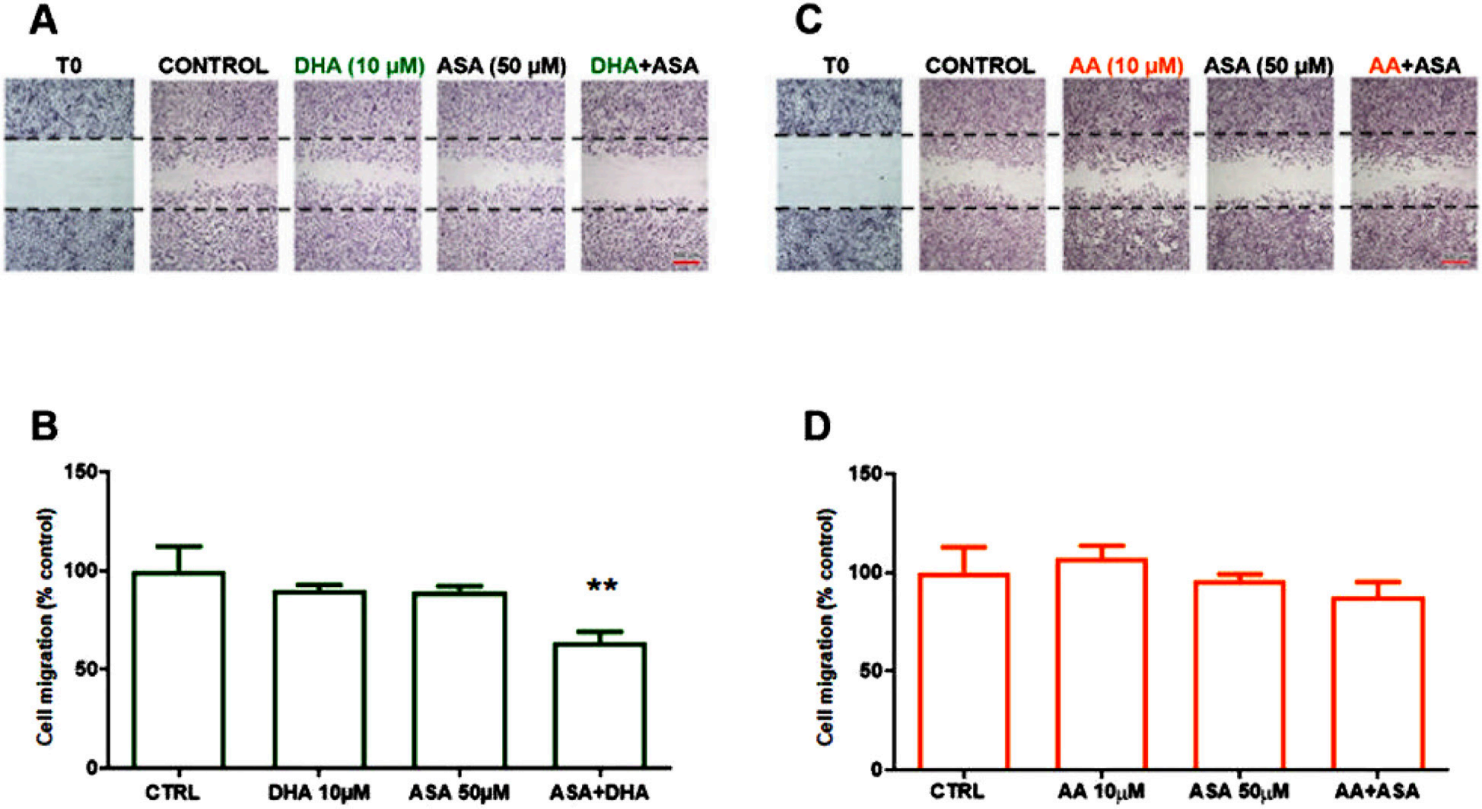
Effect of DHA ± ASA and AA ± ASA on HUVEC migration after 24h. HUVECs were grown in 24 well plates and the assay was performed in confluent cells. Cells were pre-treated with DHA (10 µM) **(A, B)** or with AA (10 µM) **(C, D)** in the presence or absence of ASA (50 µM) for 24 h. Thereafter, monolayers were wounded (t0), washed and treated as above for 16 h (t16). For each experimental condition 3 images were taken at t0 and t16; wound closure was calculated as described in Methods. **(A, C)** Representative images of a wound healing experiment in hematoxylin-eosin stained cells (scale bar: 500 µm). **(B, D)** Quantitative analysis of wound healing experiments. Data are reported as % of control (untreated cells, CTRL). Bars show the mean ± SEM of 3 **(D)** or 4 **(B)** independent experiments. One-way analysis of variance followed by Dunnet’s *post hoc* test. **p < 0.01 vs. control.

**FIGURE 4:**
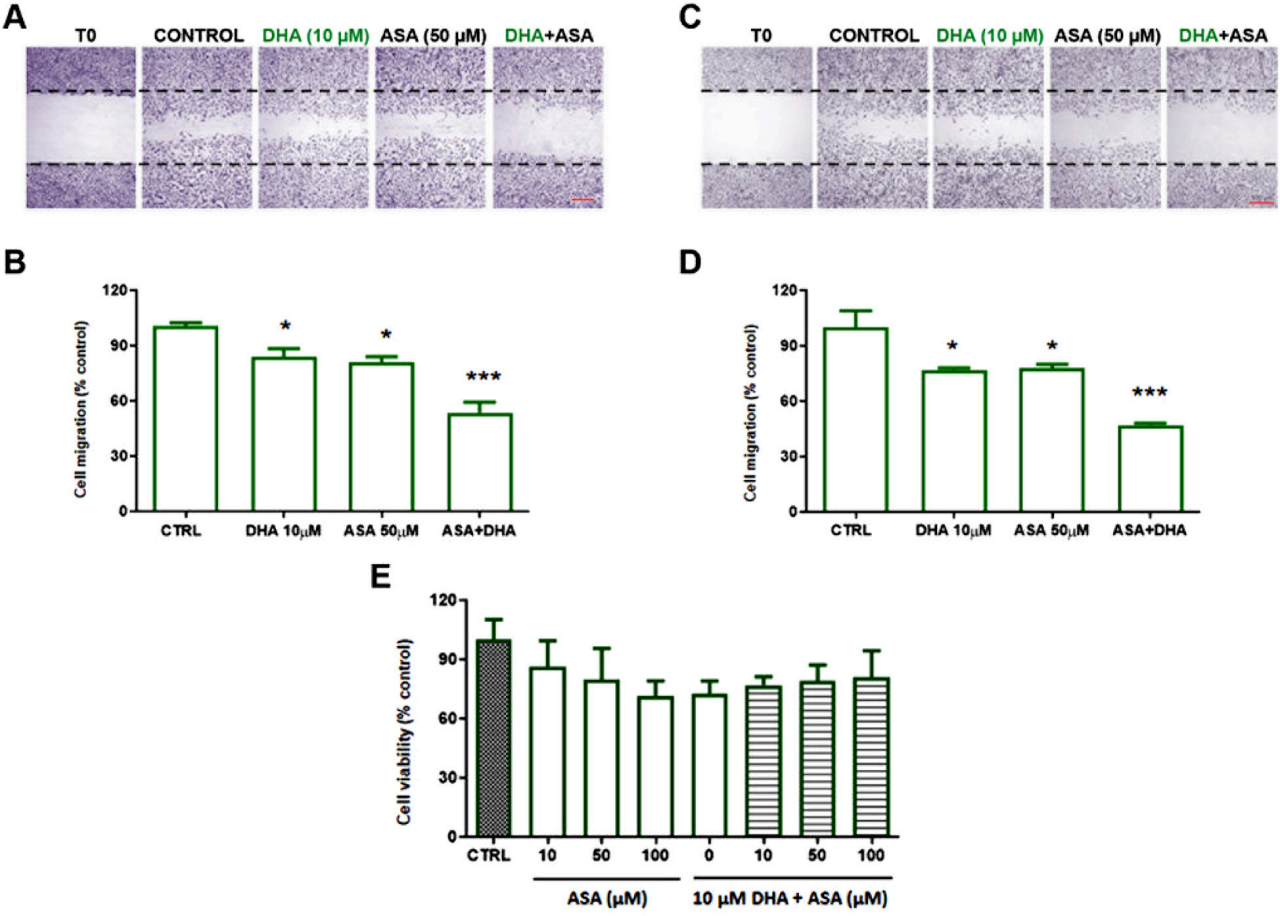
Effect of DHA ± ASA on HUVEC migration or viability after 48–72 h. HUVECs were grown in 24 well plates and the assay was performed in confluent cells. Cells were pre-treated with DHA (10 µM) in the presence or absence of ASA (50 µM) for 48 h **(A, B)** or 72 h **(C, D)**. Thereafter, monolayers were wounded (t0), washed and treated as above for 16 h (t16). For each experimental condition 3 images were taken at t0 and t16; wound closure was calculated as described in Methods. **(A, C)** representative images of a wound healing experiment in hematoxylin eosin stained cells (scale bar: 500 µm). **(B, D)** quantitative analysis of wound healing experiments. Data are reported as % of control (untreated cells, CTRL). Bars show the mean ± SEM of 3 independent experiments. One-way analysis of variance followed by Dunnet’s *post hoc* test. *p < 0.05, ***p < 0.001 vs. control. **(E)** HUVECs were grown in 96-well plates in complete medium and treated with ASA (10–100 µM) or DHA (10 µM) + ASA (10–100 µM) for 72 h. Cell viability was assessed by MTT assay and expressed as % of control (untreated cells, CTRL). Bars show the mean ± SEM of 3 independent experiments performed in quadruplicate. One-way analysis of variance followed by Dunnet’s *post hoc* test, n. s.

In the presence of 50 μM ASA, 10 µM DHA significantly inhibited HUVEC migration already after 24 h (Figures 3A, B), without affecting viability (data not shown), while neither DHA nor ASA alone resulted effective. No modifications of HUVEC migration were observed in cells treated with 10 µM AA in the presence of ASA for 24 h (Figures 3C, D). Both DHA and ASA caused a decrease in cell migration at longer incubation times of 48 and 72h, but the effect of their combination at both 48 h (Figures 4A, B) or 72 h (Figures 4C, D), exceeded the simple sum of the two separate activities.

Viability of treated cells was not different with respect to control cells at any time or concentration tested (Figure 4E).

### 2.3 17-HDHA production by endothelial cells is enhanced by incubation with ASA

**FIGURE 5:**
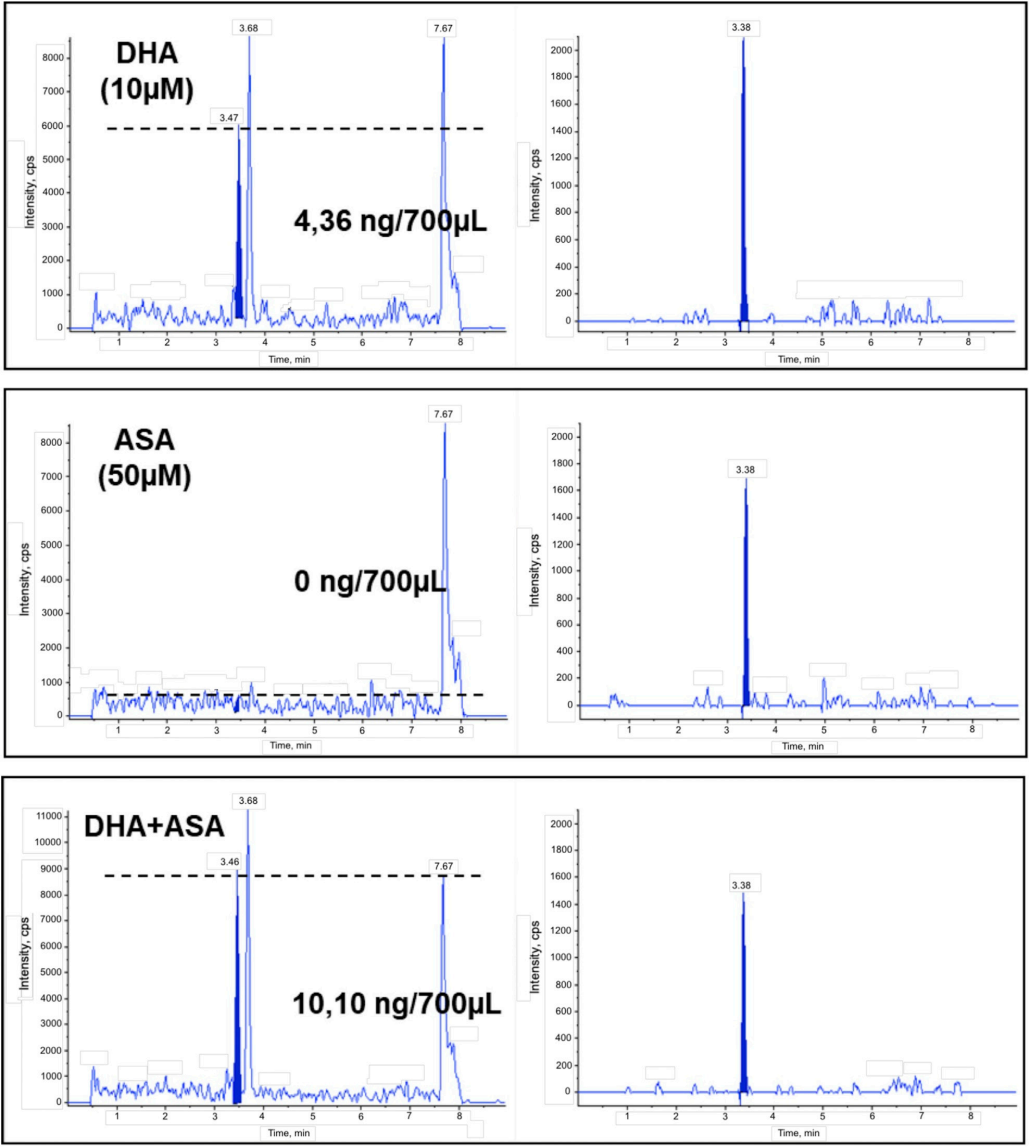
Synthesis of 17-HDHA in HUVEC treated with DHA, ASA or DHA + ASA. Representative chromatograms of the ion current for the specific transition of 17-HDHA (m/z 343 > 281) in supernatants from HUVECs treated with DHA (10 µM), ASA (50 µM) or DHA + ASA for 24 h as described in Methods (left panels). Quantitation was performed with [d^8^]15-HETE as internal standard (m/z 327 > 226, right panels) and standard curves of synthetic 17R-HDHA.

We analyzed the supernatants of cells pre-treated with DHA (10 µM), ASA (50 µM) or DHA (10 µM) + ASA (50 µM) for 24 h by liquid chromatography-tandem mass spectrometry (LC/MS/MS), showing that HUVECs biosynthesize nanomolar concentrations of 17-HDHA upon incubation with exogenous DHA, and that the production is further increased in cells treated with ASA (Figure 5).

### 2.4 The aspirin-triggered DHA metabolite 17(R)-HDHA decreased the HUVEC proangiogenic potential

**FIGURE 6:**
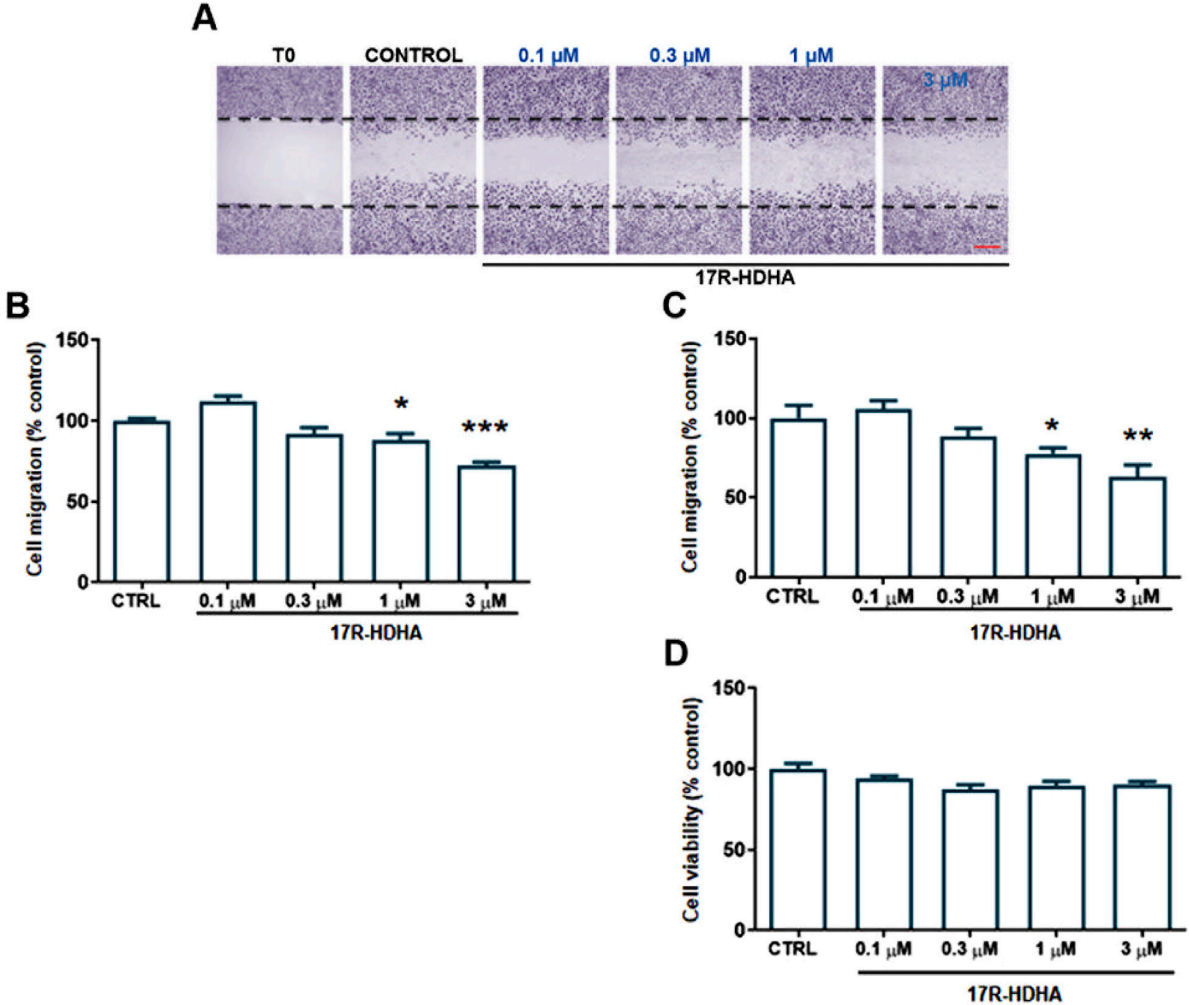
Effect of 17R-HDHA on HUVEC migration and viability after 24 or 48 h. HUVECs were grown in 24 well plates and the assay was performed in confluent cells. Cells were pre-treated with 17R-HDHA (0.1–3 µM) for 24 h **(B)** or 48 h **(A, C)**. Thereafter, monolayers were wounded (t0), washed and treated as above for 16 h (t16). For each experimental condition 3 images were taken at t0 and t16; wound closure was calculated as described in Methods. **(A)** Representative images of a wound healing experiment in hematoxylin eosin-stained cells after 48 h (scale bar: 500 µm). **(B, C)** Quantitative analysis of wound healing experiments at 24 **(B)** or 48 h **(C)**. Data are reported as % of control (untreated cells, CTRL). Bars show the mean ± SEM of 4 independent experiments. One-way analysis of variance followed by Dunnett’s *post hoc* test. *p < 0.05, **p < 0.01, ***p < 0.001 vs. control. **(D)** HUVECs were grown in 96-well plates in complete medium and treated with 17R-HDHA (0.1–3 µM) for 48 h. Cell viability was assessed by MTT assay and expressed as % of control (untreated cells, CTRL). Bars show the mean ± SEM of 3 independent experiments performed in quadruplicate. One-way analysis of variance followed by Dunnett’s *post hoc* test, n. s.

17(R)-HDHA (0.1–3 µM), showed a significant, time-dependent effect on HUVEC migration after 24–48 h (Figures 6A–C), without effects on HUVEC viability (Figure 6D).

**FIGURE 7:**
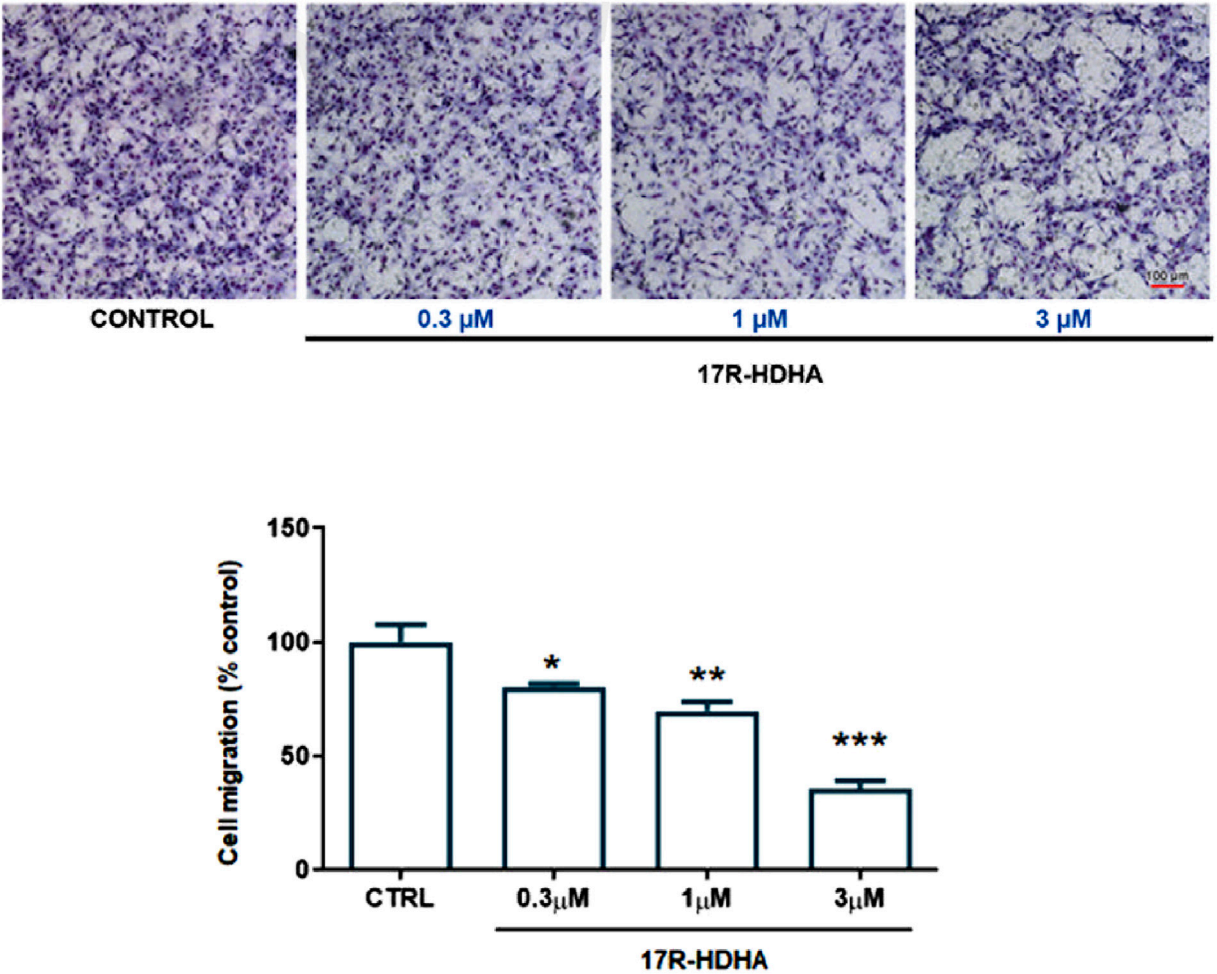
Effect of 17R-HDHA on HUVEC chemotaxis. HUVEC migration toward 15% FBS (control, CTRL) was measured in a microchemotaxis chamber in the presence or absence of increasing concentrations of 17R-HDHA (0.3–3 µM). *Upper panels*: representative images of migrated cells on the bottom of a filter membrane stained with Diff-Quick solution as detailed in Methods (scale bar: 100 µm). *Lower panel*: quantitative analysis of cell migration expressed as % of control. Bars show the mean ± SEM of 3 independent experiments performed in sextuplicate. One-way analysis of variance followed by Dunnett’s *post hoc* test. **p < 0.01, **p < 0.001 vs. control.

Furthermore, using a microchemotaxis chamber, we assessed the effect of 17(R)-HDHA on HUVEC chemotaxis, an essential step in tumor angiogenesis (Trenti et al., 2018), showing that already after 6h, 17(R)-HDHA reduced FBS-induced EC migration in a concentration-dependent manner (0.3–3 µM) (Figures 7A, B), while DHA alone resulted ineffective (data not shown).

Finally, we studied the effect of 17(R)-HDHA on tubularization, the process of organization of endothelial cells in capillary tube-like structures when cultured onto extracellular matrix proteins.

**FIGURE 8:**
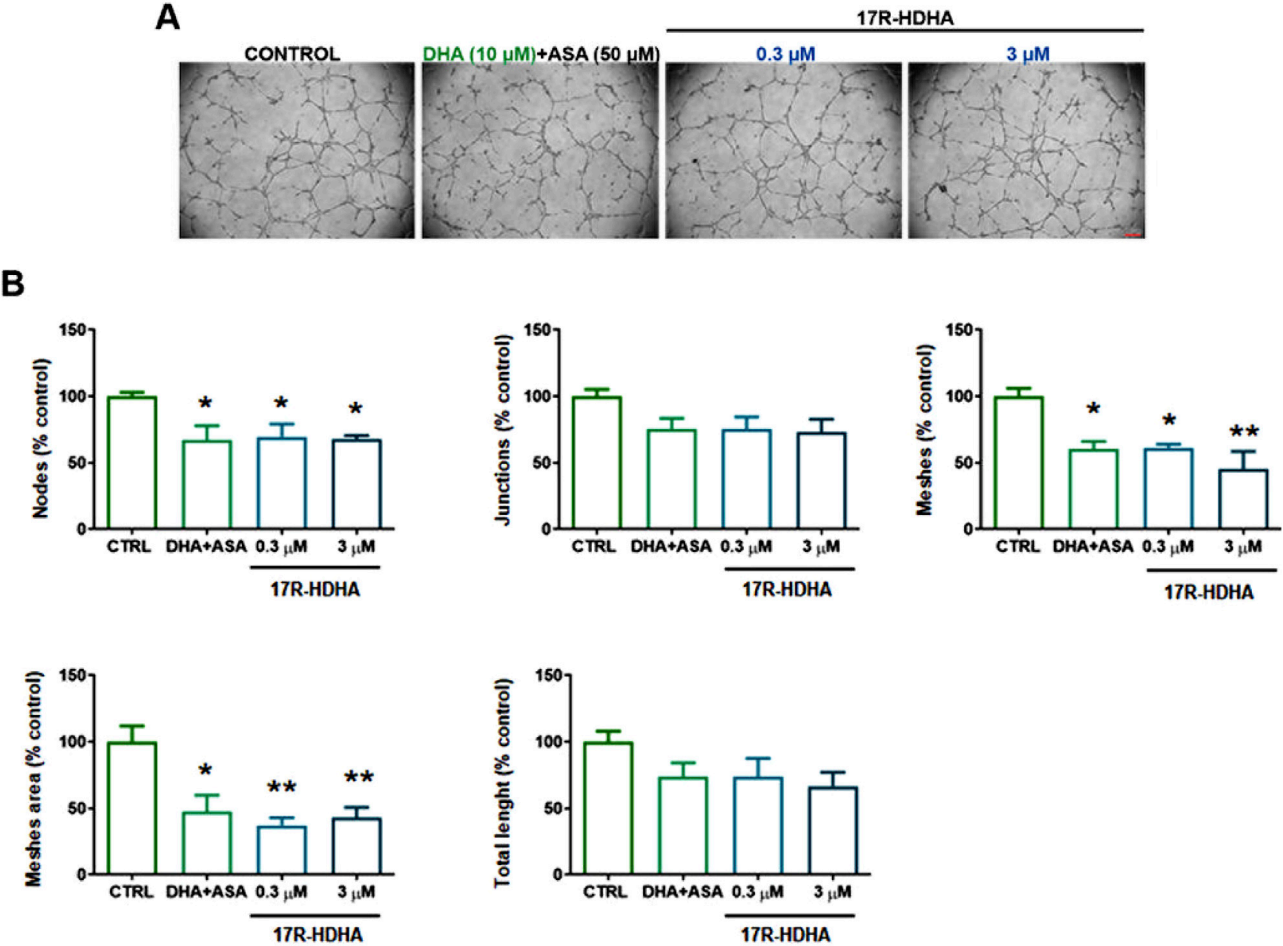
Effect of DHA in the presence of ASA or 17R-HDHA on HUVEC capillary tube formation. HUVECs were seeded onto Matrigel-coated 48-well plates in complete culture medium and treated with DHA (10 µM) +ASA (50 µM) or 17R-HDHA (0.3–3 µM) for 6 h. Complete medium with 15%FBS was taken as control (CTRL). **(A)** Representative images (scale bar: 200 µm). **(B)** Quantitative analysis of specific parameters of capillary tube as determined using Angiogenesis Analyzer (ImageJ). Bars show the mean ± SEM of 3 independent experiments expressed as % of cotrol. One-way analysis of variance followed by Dunnet’s *post hoc* test. *p < 0.05, **p < 0.01 vs. control.

In HUVECs treated with either DHA + ASA or 17(R)-HDHA for 6 h a decreased formation of specific parameters of HUVEC tubularization was observed. DHA (10 µM) in the presence of ASA (50 µM) as well as 17(R)-HDHA (0.3–3 µM) decreased nodes, meshes and mesh area. The number of junctions and the total tubule length also showed a tendency to decrease even if the difference did not reach statistical significance with respect to control cells (Figures 8A, B).

### 2.5 Aspirin-triggered DHA metabolites inhibited *in vivo* angiogenesis

To confirm *in vitro* data, we tested the effect of 17R-HDHA (3 µM) and its downstream metabolite 17R-RvD1 (50 nM) on *in vivo* angiogenesis using the Matrigel sponge model enriched with SKOV3 ovarian cells. SKOV3 cells were chosen to avoid misleading results due to potential cytotoxic effects of AT-lipid mediators on cancer cells. Indeed, *in vitro* tests of cell viability showed a higher resistance of SKOV3 compared to other ovarian cancer cells tested (CaOV and A2870) to 17R-HDHA and 17R-RvD1 treatment (data not shown).

**FIGURE 9:**
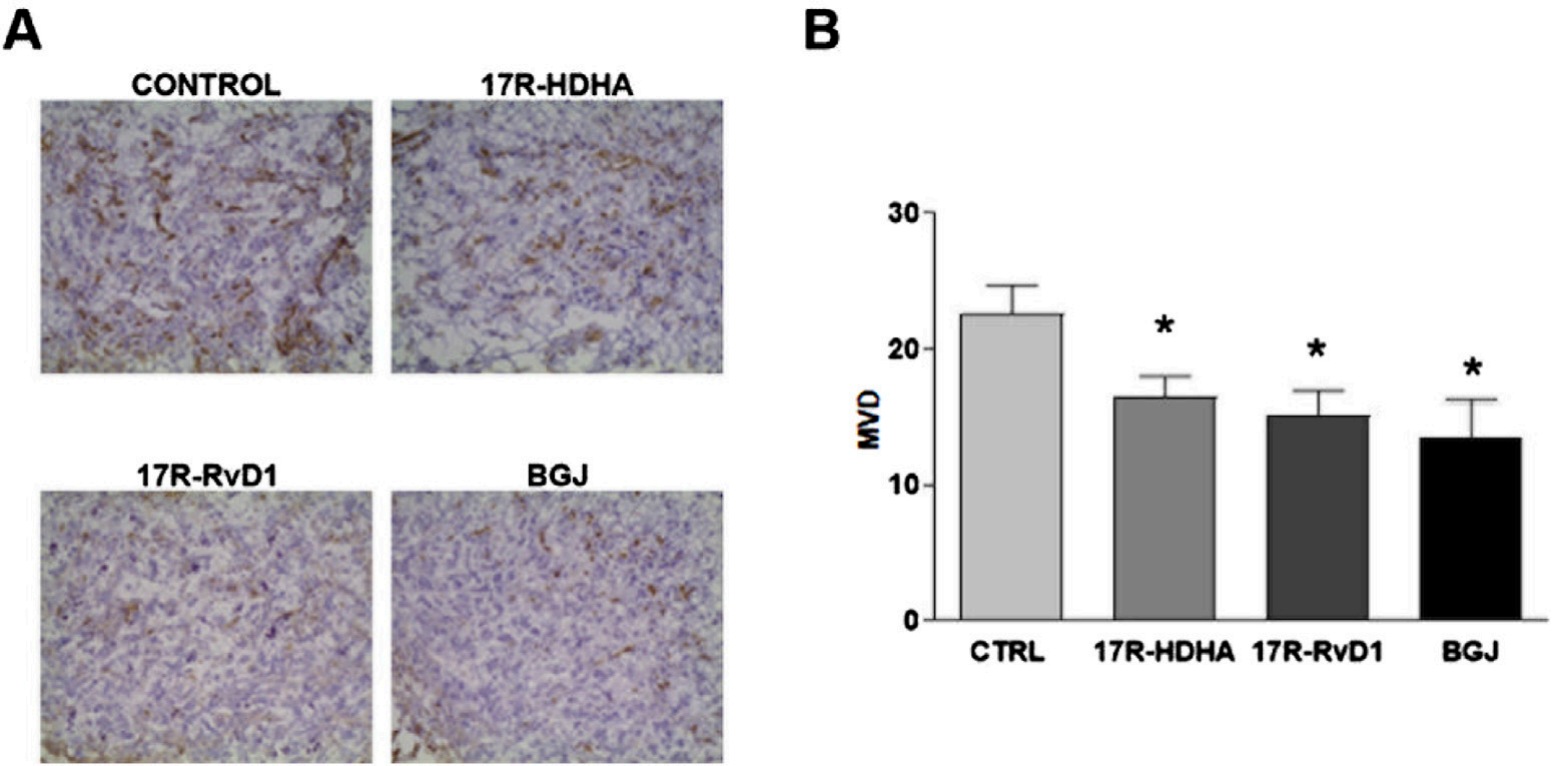
Effect of 17R-HDHA and 17R-Resolvin D1 on bFGF-induced angiogenesis *in vivo*. Vascularization of Matrigel pellets by staining with anti-CD31 mAb and calculation of microvessel density (MVD). For angiogenesis to be induced, on day 0 NOD/SCID-γ^−/−^(NSG) mice were injected s. c. with Matrigel (400 μL per injection) supplemented with bFGF (500 ng) and SKOV-3 tumor cells (5 × 10^5^cells per injection), along with 17R-HDHA (3 μM), 17R-Resolvin D1 (50 nM) or vehicle (EtOH). The pan FGFR inhibitor BGJ398 (1 μM) was used as a positive control. Seven days later, animals were killed and Matrigel pellets obtained, frozen and processed for immunohistochemical analysis. **(A)** Representative microphotographs are shown (original magnification: ×200). **(B)** Quantitative analysis of MVD. Bars show the mean ± SEM values of *n* = 5 animals per group. T-test *p < 0.05 vs. coltrol (vehicle, CTRL).

Microvessel density (MVD) was significantly reduced in mice treated with either 17R-HDHA or 17R-RvD1, when compared with controls (Figure 9), lending additional support to the results obtained *in vitro*.

## 3 Discussion

In the present study we provide initial evidence that combined treatment with DHA and ASA results in larger effects on endothelial cell migration and angiogenesis than the simple sum of the effects resulting from DHA or ASA treatments. The increased effects on angiogenesis are associated with increased amounts of 17-HDHA being produced in the presence of ASA + DHA, as the result of acetylated COX-2 acting as a 15R-lipoxygenase (Mancini et al., 1994). Administration of both exogenous 17R-HDHA and the trihydroxylated DHA derivative 17R-RvD1 were also able to modulate angiogenesis, reducing vessel formation in a model of angiogenesis *in vivo*, providing support to the hypothesis of a causal relationship between the formation of Aspirin Triggered DHA metabolites and the effects on angiogenesis.

Angiogenesis is a stepwise process requiring proliferation and migration of endothelial cells (Conway et al., 2001; Lee et al., 2021). Several studies have shown that AA and DHA may affect tumor angiogenesis in opposite ways either directly or through their oxygenated metabolites (Kang and Liu, 2013). In particular, ω3-PUFAs may act by inhibiting the production of several pro-angiogenic metabolites, including mediators derived from AA such as PGE_2_ (Spencer et al., 2009), or by directly modulating angiogenesis through the conversion into bioactive lipid mediators arising from the activities of acetylated-COX-2, LOXs and CYP450s (Gilligan et al., 2019; Kelly et al., 2024; Sulciner et al., 2018b).

ASA possesses antitumor properties that may be ascribed, at least in part, to its antiplatelet activity (Contursi et al., 2024), as well as to the ability to affect angiogenesis by inhibiting the formation of PGE_2_ (Salvado et al., 2013), a potent, COX-2 derived eicosanoid contributing to VEGF-dependent and -independent angiogenesis (Santiso et al., 2024; Xu and Croix, 2014). While ASA inhibits the biosynthesis of prostanoids both from COX-1 and COX-2, acetylation of the latter converts COX-2 into a 15-lipoxygenase, capable of metabolizing both AA and DHA into 15(R)-hydroxyeicosatetraenoic acid (15R-HETE) and 17R-HDHA, respectively (Mancini et al., 1994; Serhan et al., 2002); both compounds also serves as the substrate for the 5-LOX-dependent formation of specific Aspirin-Triggered (AT)-lipid mediators possessing protective effects in inflammatory diseases (Romano et al., 2015; Serhan et al., 2002). A limited number of studies investigated the antiangiogenic properties of AT-lipid mediators, mainly focusing on AA-metabolites (Cezar-De-Mello et al., 2006; 2008) or RvD1 (Maisto et al., 2020) but little is known about the potential role of 17R-HDHA.

Normal plasma concentrations of DHA, assessed by GC/FID or GC/MS in humans, range between 70 and 160 μg/mL, that is 150–500 µM (Buchanan et al., 2021; Lv and Yang, 2012). Nevertheless, free fatty acids account for a small fraction of plasmatic fatty acids, with concentrations ranging from 200 to 600 µM in total, that is between 2 and 6 µM for DHA (Yli-Jama et al., 2002), values that we showed can increase by 2-3 folds upon DHA supplementation with either a fish-rich diet or fish-oil capsules (Visioli et al., 2003). We therefore assessed the effects of ω3-PUFAs at the concentration of 10 μM, and their potential positive interaction with ASA on collective HUVEC migration using a wound healing migration assay, showing that both treatments decrease EC migration, without affecting cell viability. Moreover, the effect observed in the presence of both ASA and DHA was more than additive, suggesting that ASA may potentiate the activity of DHA or vice-versa. Acetylation of COX-2 by ASA prevents the formation of pro-angiogenic PGE_2_ prostanoids in HUVECs (Mulligan et al., 2010; Tamura et al., 2006), but in the presence of DHA the acetylated enzyme may still synthesize 17R-HDHA that can either act on its own or be further metabolized by 5-LO into 17R-resolvins (Serhan and Levy, 2018).

Detection of 17-HDHA in supernatants from HUVECs incubated with DHA is in agreement with results previously obtained using vascular tissues (Chatterjee et al., 2017), and the increased amounts observed in the presence of ASA suggest that acetylated COX-2 in HUVECs may contribute to the final production of 17-HDHA. It must be noted that we did not carry out chiral analysis of the observed hydroxylated metabolite of DHA, and therefore it is possible that in the presence of DHA alone the stereochemistry of the 17-hydroxyl may be S or a combination of S and R, while the additional formation of 17-HDHA observed in the presence of ASA could be referred to the formation of the R epimer, according to the stereochemistry of the product resulting from acetylation of COX-2.

17S-HDHA, the epimer of 17R-HDHA, is metabolized within 4 h to generate Rv-D1 and this conversion *in vivo* is mainly mediated by transcellular metabolism involving interaction between inflammatory (e.g. macrophage or neutrophils) and endothelial cells (Serhan et al., 2002; Chatterjee et al., 2017). Although it is known that R epimers resist rapid inactivation by oxidoreductases and have longer half-lives with respect to the S epimers, limited evidence is available about the *in vivo* biological activity of 17R-HDHA. The assessment of the direct activity of 17R-HDHA on HUVECs, showed that this metabolite is clearly effective in modulating their pro-angiogenic potential, an effect that was confirmed also by *in vivo* administration in an animal model of angiogenesis. While 17R-RvD1 was also able to affect microvessel formation *in vivo* (and at lower concentrations), it must be noted that *in vivo* formation of di- and tri-hydroxylated DHA derivatives (such as the 17R-RvD1) has been questioned over the last few years (O’Donnell et al., 2023; Schebb et al., 2022). Undetectable levels of SPMs were reported under different conditions, including supplementation with high doses of DHA and *in vivo* challenge with LPS in humans (Skarke et al., 2015), while *in vitro* formation of monohydroxylated DHA derivatives in human leukocytes vastly exceeded that of (if any) di- and tri-hydroxylated metabolites (Kahnt et al., 2023).

Indeed, we have been able to consistently report the *in vivo* formation of 14-hydroxy and 17-hydroxy-docosahexaenoic acid in different pathological conditions (Teopompi et al., 2019; Terranova et al., 2022), and we believe that monohydroxy derivative of DHA may play an important role in mediating the biological effects of DHA and DHA supplementation. Almost 40 years ago, we reported that the 12-lipoxygenase derived DHA metabolite, namely the 14-hydroxy-docosahexaenoic acid, is a potent receptor antagonist of Thromboxane A2 (Croset et al., 1988), and the present results support another relevant activity for a DHA monohydroxy-derivative.

It must be noted that our study has limitations: while the effects observed on isolated endothelial cells may support the activity observed *in vivo,* the specific contribution of direct effects on these cells and indirect systemic effects will require additional investigation. The use of the Matrigel sponge also may have a limited relevance with respect to specific tumor angiogenesis, but it must be noted that this experimental model is still widely accepted for the evaluation of the angiogenic potential or anti-angiogenic activity of various compounds in experimental animals (Benton et al., 2014; Kastana et al., 2019).

Overall, the data presented are showing that DHA and ASA may synergistically affect endothelial cell migration and angiogenesis, and that 17R-HDHA can contribute to these activities, as also shown by its ability to affect invasion and microvessels formation *in vivo*. While additional research is necessary to establish the molecular mechanisms of these activities, the evidence obtained may contribute to explain the beneficial effects of aspirin and ω-3 FA in the context of cancer.

## 4 Materials and methods

### 4.1 HUVEC isolation and culture

Human umbilical vein endothelial cells (HUVECs) were isolated as previously published (Bolego et al., 2006). Briefly, umbilical cords were collected after delivery, from full-term normal pregnancies at the Obstetrics and Gynaecological Unit of Padua University Hospital. The donors gave their informed consent, and the collected cords were non-identifiable. The procedure was approved by the local Ethics Committee (Comitato Etico per la Sperimentazione Clinica della Provincia di Padova). Each HUVEC preparation was derived from at least three donors and cells were pooled after isolation. Cells were grown in medium M199 (Thermo Fisher Scientific, Waltham, MA, United States) supplemented with 15% fetal calf serum (FCS, Thermo Fisher Scientific), gentamicin (40 μg/mL, Thermo Fisher Scientific), endothelial cell growth supplement (ECGS, 100 μg/mL), and heparin (100 UI/mL, Sigma-Aldrich, Saint Louis, MO) at 37°C in a humidified 5% CO_2_ atmosphere. HUVECs were identified by their morphology and the expression of CD31-related antigen and used for experiments from passages 2 through 6.

### 4.2 MTT assay

HUVECs were seeded in complete medium at different cell densities considering the duration of the treatment (2 × 10^4^, 1.5 × 10^4^ or 10^4^ cells for 24, 48 and 72 h of treatment, respectively) in 96-well plates. The next day, cells were treated in fresh medium containing DHA or AA (Cayman Chemical, Ann Arbor, United States), or with ASA in the presence or absence of DHA (Sigma-Aldrich), as detailed in the Results section. Selected experiments were performed by treating cells with increasing concentrations of 17R-HDHA (Cayman Chemical) for 72 h. Four hours before the end of incubation, 10 μL of 3-[4,5 dimethylthiazol-2-yl]-2,5 diphenyltetrazolium bromide (MTT, 5 mg/mL in phosphate-buffered saline, Sigma Aldrich) was added to each well. Then, the medium was removed and formazan crystals were dissolved in 100 μL dimethylsulfoxide. MTT reduction was quantified by measuring light absorbance with a Wallac Victor^2^ plate reader (PerkinElmer, Waltham, MA, United States) at 570–630 nm. Background absorbance values from control wells (cell-free media) were subtracted. Cell viability is expressed as percent of controls.

### 4.3 Chemotaxis assay

Chemotaxis experiments were performed in a 48-well microchemotaxis chamber (Neuro Probe, Gaithersburg, MD, United States) using 8 μm polyvinylpyrrolidone-free polycarbonate filters coated with 10 μg mL^−1^ of collagen (rat tail, Roche, Basel, Switzerland). Upper chambers were filled with 50 μL HUVEC suspension (1.6 × 10^5^ cells per mL in M199 supplemented with 1% FBS and 100 UmL^−1^ heparin). Lower chambers were filled with 28 μL of complete M199 supplemented with 100 UmL^−1^ heparin in presence of 15% FBS plus 100 μg mL^−1^ ECGS. Cell migration towards complete medium with 15% FBS was taken as control. For the evaluation of the basal motility, M199 supplemented with 1% FBS and 100 UmL^−1^ heparin was added in the lower chamber. 17R-HDHA (0.3–3 µM) was added both in the upper and lower compartment. After 6 h of incubation at 37°C, the non-migrated HUVECs on the upper surface of the filter were removed by scraping. The cells that migrated to the lower side of the filter were stained with Diff-Quick stain (vWR Scientific Products, Bridgeport, NJ). Five images per well were acquired with a phase contrast inverted microscope (Nikon Eclipse Ti, Shinagawa, Tokyo, Japan) equipped with a digital camera using a ×20 objective. Cells were counted using a Cell Counter plugging developed by ImageJ version 1.47 software (National Institute of Health, United States). Each experimental condition was performed in sextuplicate. Results are expressed as percent of control.

### 4.4 Collective migration assay

HUVECs were seeded in complete medium at different cell densities considering the duration of the treatment (2 × 10^5^, 1.5 × 10^5^ or 10^5^ cells for 24, 48 and 72 h of treatment, respectively) in 12-well plates. The next day the media was replaced with fresh complete medium containing AA or DHA in the presence or absence of ASA for up to 72 h as detailed in the Results section. Selected experiments were performed treating cells with 17R-HDHA for 24 or 48 h. After that, one scratch was made and cells were incubated in fresh medium containing the tested compounds for additional 16 h. At the end of the experiment, three images of each well were acquired with a phase contrast inverted microscope (Nikon Eclipse Ti) equipped with a digital camera using a ×4 objective immediately after the scratch was made (time 0) and after 16 h of incubation. The wound area of each image was measured using ImageJ, and the average wound area of three images was determined for each sample. Quantitative analysis of cell migration was performed as the percentage of area change using the following formula: %change = [(average wound area at t0 − average wound area at t16) ÷ average wound area at t0] × 100. Values are expressed as % change from control (untreated cells).

### 4.5 Capillary-like tube formation assay

HUVECs (2 × 10^4^ cells) were plated onto a thin layer (120 µL) of a basement membrane matrix (Matrigel™, Corning Corp., Corning, NY, United States) in 48-well plates, and incubated at 37°C for 6 h in complete cell culture medium in the presence or absence of test compounds as indicated in the Results. Complete cell culture medium with 15% FBS was taken as control. Three images per well were acquired with a phase contrast inverted microscope (Nikon Eclipse Ti) equipped with a digital camera using a ×4 objective. Images were analyzed using Angiogenesis Analyzer, a plugin developed for the ImageJ software. The data on dimensional parameters (total tubule length) and topological parameters (number of junctions, nodes and meshes, and total mesh area) of the capillary-like network (Trenti et al., 2017) were analyzed in all the images obtained from control and treated wells. Data are expressed as percent change from controls.

### 4.6 Biosynthesis of proresolving lipid mediators by LC-MS-MS

HUVECs (2 × 10^5^ cells) were seeded in complete medium in 12-well plates and treated with DHA or ASA or DHA + ASA for 24 h. At the end of the experiment, cell medium was harvested and analyzed by Liquid chromatography-tandem mass spectrometry as previously published (Teopompi et al., 2019) with modifications. Briefly, samples were centrifuged to remove any cellular material. 50 μL of internal standard ([d^8^]15-HETE, Cayman Chemical) were added to 700 μL of sample and immediately applied to polymeric SPE cartridges (Strata-X, 33 μm Polymeric Reversed Phase; Phenomenex, Torrance, CA) that had been preconditioned with 1 mL methanol and 1 mL water. After washing with 1 mL of water, the hydroxy-fatty acids were eluted using 400 μL of a acetonitrile:methanol (65%:35%). Samples were evaporated to dryness by centrifugation under vacuum (SpeedVac; Thermo Scientific, Waltham, MA) and reconstituted in 100 μL of a solution 70% (v/v) phase A (water, acetic acid 0.05%, pH: 5.7) and 30% (v/v) phase B (65% acetonitrile and 35% methanol). 10 μL of each sample were injected in an HPLC (Agilent 1,100) equipped with a reverse phase column (Kinetex 5 µm C18, 50 × 2.1 mm, Phenomenex, Castel Maggiore, BO, Italy). The column was eluted with a linear gradient from 30% to 100% solvent B over 9 min. The effluent from the high-performance liquid chromatography (HPLC) column was directly infused into the electro spray source of an API4000 triple quadrupole (ABSciex, Framingham, MA) operated in negative ion mode. Quantitation was performed using standard curves obtained with synthetic standard (17R-HDHA, Cayman Chemical) and stable isotope dilution.

### 4.7 *In vivo* angiogenesis

#### 4.7.1 Animals and treatment

All procedures involving animals and their care conformed to institutional guidelines that comply with national and international laws and policies (EEC Council Directive 86/609, OJ L 358, 12 December 1987) and were authorized by the Italian Ministry of Health (Authorization n. 129/2017-PR). Animal studies are reported in compliance with the ARRIVE guidelines (Kilkenny et al., 2010; McGrath et al., 2015). During *in vivo* experiments, animals in all experimental groups were examined daily for a decrease in physical activity and other signs of disease or drug toxicity. Six to eight-week-old female NOD/SCID-γ^−/−^ (NSG) mice were purchased from Charles River Laboratories (Wilmington, MA, United States) and housed in our specific pathogen-free animal facility in Allentown IVC cages (floor area 542 cm^2^) with a maximum of six mice per cage. All mice received water and food *ad libitum* and were kept under a 12 h light/dark cycle in a well-ventilated room at an approximate temperature of 22°C. Mice acclimatized for a minimum of 7 days and a maximum of 15 days before being randomly assigned to treatment or vehicle groups.

The Matrigel sponge model of *in vivo* angiogenesis introduced by Albini et al. (1994), Passaniti et al. (1992) was used. For angiogenesis to be induced, NSG mice were randomly divided into four groups of five animals each and injected s. c. into both flanks with 400 μL Matrigel supplemented with 500 ng bFGF and SKOV-3 tumor cells (5 × 10^5^ cells per injection), along with either 17-R-HDHA (3 µM), 17-R-RvD1 (50 nM, Cayman Chemical) or vehicle (EtOH). The pan FGFR inhibitor BGJ398 (Selleck Chemicals GmbH, Cologne, Germany) was used as positive control (Rezzola et al., 2021). Seven days after injection, mice were anaesthetized with isoflurane/oxygen and killed via cervical dislocation, and Matrigel pellets (two pellets per mouse) were collected.

#### 4.7.2 Evaluation of MVD

Four-μm-thick frozen sections of Matrigel pellets were processed for immunohistochemistry as previously reported (Nardo et al., 2011). Microvessels were stained by rat anti-CD31 mAb (1:50 dilution; Becton Dickinson, East Rutherford, NJ, United States); immunostaining was performed using the avidin–biotin–peroxidase complex technique and 3–3′ diaminobenzidine as chromogen (Vector Laboratories, Burlingame, CA, United States), and the sections were then lightly counterstained with Mayer’s haematoxylin. Parallel negative controls, obtained by replacing primary Abs with PBS, were run. Microvessel density (MVD) was quantified by screening the CD31-stained sections for the areas of highest vascularity. The number of fields analyzed varied between 5 and 10 per sample, depending on the sample size. Images were collected at a total magnification of ×200. For each animal, the mean value of replicates was used for statistical analysis; five animals per group were analyzed.

## 5 Statistical analysis

All data represent the results of at least three independent experiments. Results are expressed as mean ± standard error of the mean (SEM). GraphPad Prism Software, version 8.4 (San Diego, CA, United States) was used for plotting of the data and statistical analysis. Data were analyzed by one-way ANOVA followed by Dunnett’s *post hoc* test or T-test as detailed in the figure legends. Differences were considered statistically significant for p < 0.05.

## Data Availability

The raw data supporting the conclusions of this article will be made available by the authors, without undue reservation.
